# Salt-Dependent RNA Pseudoknot Stability: Effect of Spatial Confinement

**DOI:** 10.3389/fmolb.2021.666369

**Published:** 2021-04-13

**Authors:** Chenjie Feng, Ya-Lan Tan, Yu-Xuan Cheng, Ya-Zhou Shi, Zhi-Jie Tan

**Affiliations:** ^1^Key Laboratory of Artificial Micro and Nano-structures of Ministry of Education, Center for Theoretical Physics, School of Physics and Technology, Wuhan University, Wuhan, China; ^2^Research Center of Nonlinear Science, School of Mathematics and Computer Science, Wuhan Textile University, Wuhan, China

**Keywords:** coarse-grained model, RNA pseudoknot, spatial confinement, salt effect, stability

## Abstract

Macromolecules, such as RNAs, reside in crowded cell environments, which could strongly affect the folded structures and stability of RNAs. The emergence of RNA-driven phase separation in biology further stresses the potential functional roles of molecular crowding. In this work, we employed the coarse-grained model that was previously developed by us to predict 3D structures and stability of the mouse mammary tumor virus (MMTV) pseudoknot under different spatial confinements over a wide range of salt concentrations. The results show that spatial confinements can not only enhance the compactness and stability of MMTV pseudoknot structures but also weaken the dependence of the RNA structure compactness and stability on salt concentration. Based on our microscopic analyses, we found that the effect of spatial confinement on the salt-dependent RNA pseudoknot stability mainly comes through the spatial suppression of extended conformations, which are prevalent in the partially/fully unfolded states, especially at low ion concentrations. Furthermore, our comprehensive analyses revealed that the thermally unfolding pathway of the pseudoknot can be significantly modulated by spatial confinements, since the intermediate states with more extended conformations would loss favor when spatial confinements are introduced.

## Introduction

RNAs have been shown to perform many crucial biological functions, including the regulation of gene transcription and translation and the catalysis of RNA splicing (Serganov and Patel, 2007; Sashital and Doudna, 2010). An RNA pseudoknot is a very common RNA structure motif and can play important roles in many cellular functions (e.g., stimulating programmed−1 ribosomal frameshifting), which are directly related to the folding stability and conformational changes of the pseudoknot (Giedroc et al., 2000; Staple and Butcher, 2005). Accordingly, to determine 3D structures and stability of RNA pseudoknots is essential to understand and utilize their related functions (Giedroc et al., 2000; Giedroc and Cornish, 2009; Wang et al., 2015a).

Although experimental methods, such as X-ray crystallography, nuclear magnetic resonance spectroscopy, and cryo-electron microscopy, can be used to determine the structures of RNAs including pseudoknots, the structures in Protein Data Bank (PDB; https://www.rcsb.org) are still limited due to the high cost of the experimental measurements (Hajdin et al., 2010; Rose et al., 2011; Shi et al., 2014b; Schlick and Pyle, 2017). To complement the experiments, some computational models/methods (e.g., FARNA, MC-Fold/MC-Sym, Vfold, iFoldRNA, 3dRNA, RNAComposer, SimRNA, oxRNA, HiRE-RNA, and pk3D) have been developed for predicting RNA 3D structures (Cao and Chen, 2005; Ding et al., 2008; Parisien and Major, 2008; Zhang et al., 2009; Das et al., 2010; Popenda et al., 2012; Zhao et al., 2012; He et al., 2013, 2015, 2019; Kim et al., 2014; Liwo et al., 2014, 2020; Sulc et al., 2014; Cragnolini et al., 2015; Wang et al., 2015a,b; Boniecki et al., 2016; Dawson et al., 2016; Li et al., 2016, 2018; Tan et al., 2019). Most of these models/methods are primarily designed to predict folded structures and cannot predict the stability of RNAs, especially in ion solutions (Shi et al., 2014b; Dawson et al., 2016; Schlick and Pyle, 2017), whereas the structural stability of RNAs can be very sensitive to ion conditions due to their polyanionic nature (Das et al., 2005; Draper et al., 2005; Tan and Chen, 2007, 2011; Qiu et al., 2010; Lipfert et al., 2014; Wang et al., 2018, 2020; Meng et al., 2020). Recently, to predict the 3D structures, stability, and flexibility of RNAs in ion solutions from sequences, we have developed a new coarse-grained (CG) model, and the model has been validated for extensive RNA hairpins, kissing complex, and pseudoknots over a wide range of monovalent/divalent ion concentrations (Shi et al., 2014a, 2015, 2018; Jin et al., 2018, 2019).

Furthermore, RNAs are generally residing in crowded cellular microenvironment with many other macromolecules, and the volume percentage of macromolecules could take up to ~40% (Leamy et al., 2016). Recent experiments showed that spontaneous folding of RNAs and proteins in crowded environment can be substantially different from that observed in *in vitro* experiments, usually conducted only in the presence of small molecular weight buffers (Kilburn et al., 2013, 2016; Paudel and Rueda, 2014; Leamy et al., 2017; Yamagami et al., 2018). For example, small angle X-ray scattering (SAXS) measurements on ribozymes in the presence of polyethylene glycol indicated that molecular crowding can apparently stabilize the compaction of folded structures and favor the transition from unfolded state to folded state at low ion concentrations (Kilburn et al., 2010, 2013, 2016). Thermal denaturation and SAXS studies also showed that crowding agents can promote the tRNA folding cooperativity by stabilizing its tertiary structure in physiological ion concentrations (Leamy et al., 2017; Yamagami et al., 2018). Meanwhile, several theoretical studies have also been performed to investigate how molecular crowding influences RNA stability (Denesyuk and Thirumalai, 2011; Tan and Chen, 2012; Dupuis et al., 2014; Feig et al., 2017). For example, Denesyuk and Thirumalai used a three-interaction-site CG model to simulate the switch from hairpin to pseudoknot conformations in the human telomerase RNA under different crowded environments and showed that crowders could enhance the stability of pseudoknot relative to hairpin state through reducing the population of extended conformations in the unfolded state (Denesyuk and Thirumalai, 2011). Due to the salt-dependent compactness of unfolded states of RNAs, the crowding effect could be very sensitive to ion conditions. However, previous works for RNAs under crowded environment were mainly focused on high salt conditions, and thus the electrostatic interactions are minimally present. Very recently, Yu et al. developed a new statistical mechanical treatment to predict the effect of molecular crowding on ion–RNA interactions, but the model is limited for the folded RNA conformations and could not be applicable for RNA folding (Yu et al., 2016). Therefore, how macromolecular crowding impacts the salt-dependent 3D structures and stability of RNAs is still elusive.

In this study, we used a typical RNA pseudoknot as a paradigm to systematically investigate the effect of spatial confinement on the 3D structures, stability, and unfolding pathways of RNAs in ion solutions. First, we employed our CG model to predict 3D structures and stability of the RNA pseudoknot under various spatial confinements over a wide range of ion concentrations. Afterward, we examined the effect of spatial confinement on the salt-dependent 3D structures and stability for the RNA pseudoknot. Finally, we made comprehensive analyses on the effect of spatial confinement on the thermally unfolding pathways for the RNA pseudoknot in different ion solutions.

## Materials and Methods

### The CG Structure Model and Force Field

To predict the 3D structures and stability of RNAs in various ion solutions, we have developed a new CG model with involving the effects of ions (Shi et al., 2014a, 2015, 2018; Jin et al., 2018, 2019). In the model, each nucleotide is represented by three beads, which represent the three atom groups: phosphate group (P), sugar ring (C), and base (N), respectively. The P and C beads within the backbone are placed at the P and C4′ atoms, respectively, and the N beads are placed at N9 atoms for purine or N1 atom for pyrimidine (Shi et al., 2014a). The interactions between the CG beads are composed by eight energy terms. Namely, for a given RNA conformation, the effective potential energy (*U*) is given by:

(1) $$\begin{array}{l} U=U_{b}+U_{a}+U_{d}+U_{exc}+U_{bp}+U_{bs}+U_{cs}+U_{el}, \end{array}$$

where the first three terms are bonded potentials, and the remaining terms are non-bonded potentials to describe various pairwise non-bonded interactions. The function forms and the determination of the parameters for the eight terms in Equation (1) have been described in detail in Shi et al. (2014a) and can also be found in Supplementary Material. The energy terms were described very briefly as follows.

The bond length energy *U*_*b*_, bond angle energy *U*_*a*_, and dihedral energy *U*_*d*_ account for the connectivity and angular rotation for an RNA chain, and their parameters have two sets: Para_nonhelical_ for single strands/loops in RNA folding process and Para_helical_ for helical stems only in structure refinement, which are derived from the statistical analysis of the 3D structures of RNAs in the PDB (https://www.rcsb.org) (Rose et al., 2011). The *U*_*exc*_ in Equation (1) represents excluded volume interactions between two CG beads, and the P, C, and N beads are treated as spheres with van der Waals radii (*r*) of 1.9 Å, 1.7 Å, and 2.2 Å, respectively. The *U*_*bp*_, *U*_*bs*_, and *U*_*cs*_ in Equation (1) are the base-pairing (between Watson–Crick and wobble base pairs), base-stacking (between nearest-neighbor base pairs), and coaxial stacking (between two discontinuous neighbor helices) interactions, respectively, and the strength of them was derived from sequence-dependent thermodynamic parameters and the corresponding experimental data (Xia et al., 1998; Spasic et al., 2018). The last term *U*_*el*_ in Equation (1) is an electrostatic potential corresponding to electrostatic interactions between phosphate groups (a charge of –*e* for each P bead at its center), which are ignored by most existing predictive models for RNA 3D structures (Hajdin et al., 2010; Rose et al., 2011; Shi et al., 2014b; Schlick and Pyle, 2017). The *U*_*el*_ is taken into account through the combination of the Debye–Hückel approximation, the concept of counterion condensation (CC) (Manning, 1978), and the tightly bound ion (TBI) model (Tan and Chen, 2007, 2010, 2011), and the potential can well-capture the contribution of monovalent/divalent ions to RNA 3D structures (Shi et al., 2015; Jin et al., 2019).

### Material and Spatial Confinement

**Figure 1:**
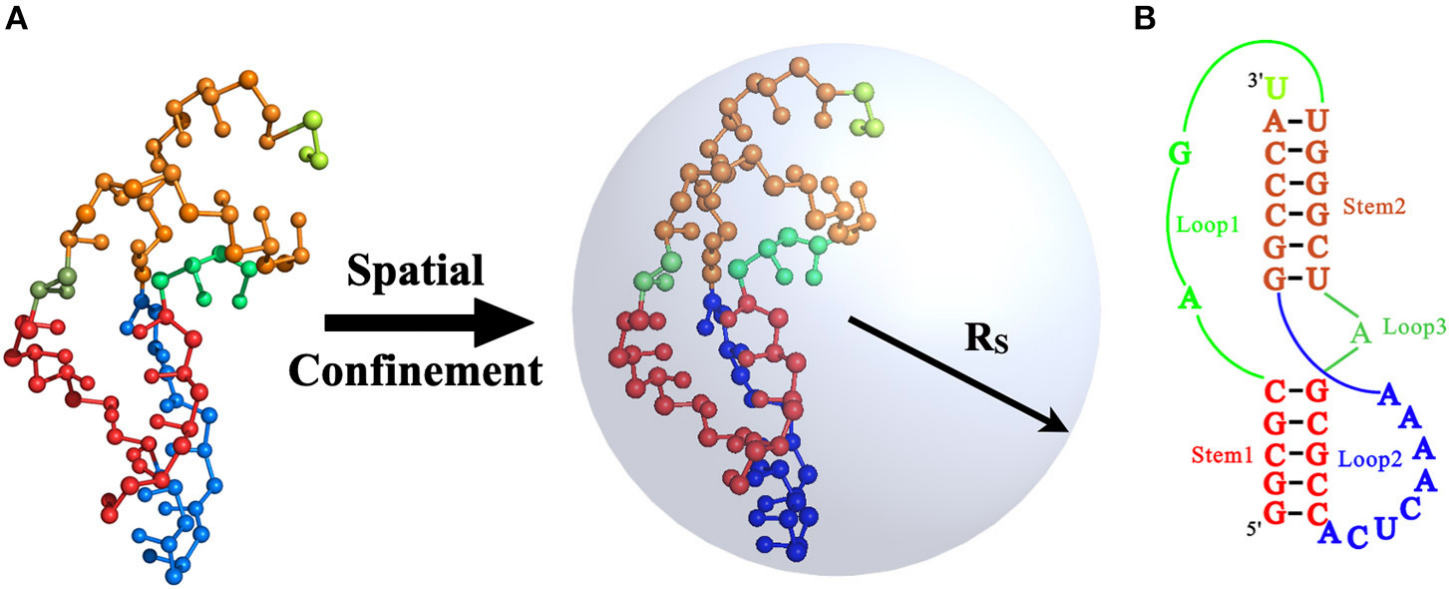
**(A)** The schematic illustration for the mouse mammary tumor virus (MMTV) pseudoknot under the spatial confinement of radius *R*_S_ and the 3D structure of the pseudoknot (PDB code: 1rnk; https://www.rcsb.org) in our coarse-grained (CG) representation. **(B)** The secondary structure for the MMTV pseudoknot consisting two stems and three loops. The corresponding secondary elements in **(A,B)** are in same colors: Stem 1 (red), Stem 2 (orange), Loop 1 (green), Loop 2 (blue), Loop 3 (dark green), and the 3′ dangling nucleotide (light green). The 3D structures in **(A)** are shown with the PyMol (http://www.pymol.org).

In this work, we used the 34-nucleotide RNA pseudoknot from mouse mammary tumor virus (MMTV; PDB code: 1rnk; sequence: 5′-GGCGCAGUGGGCUAGCGCCACUCAAAAGGCCCAU-3′; https://www.rcsb.org) as a model RNA to investigate the crowding effect on RNA folding, considering the biological role of the MMTV pseudoknot in stimulating programmed −1 ribosomal frameshifting in the ribosome tunnel (Shen and Tinoco, 1995). The sequence and experimental structures are shown in Figure 1. As shown in Figure 1, the pseudoknotted structure is formed when a sequence of nucleotides within a single-stranded loop region forms base pairs with a complementary sequence outside that loop, and it includes two stems (Stem 1 and Stem 2) and three loops (Loop 1, Loop 2, and Loop 3).

Although the interactions between macromolecule crowders and RNAs could be very complex, experimental and theoretical studies have shown that when the crowding particles are much bigger and heavier than the target RNA (e.g., RNA pseudoknots in the ribosome tunnel), the macromolecular crowding effects can be approximated by a spatial confinement, the shape and dimensions of which would depend on the crowding concentration (Cheung et al., 2005; Mittal and Best, 2008; Zhou et al., 2008; Qin and Zhou, 2014; Hori et al., 2016; Feig et al., 2017). For example, Cheung et al. used a spherical cavity to mimic the effect of crowding and obtained the mapping between crowding and effective confinement (Cheung et al., 2005):

(2) $$\begin{array}{l} R_{s}={\left(\frac{4\pi }{3\varphi_{c}}\right)}^{\frac{1}{3}}R_{c}, \end{array}$$

where *R*_S_ is the radius of spherical pore, and *R*_c_ is the radius of the macromolecular crowding agents. φ_*c*_ in Equation (2) is the volume fraction of crowding agents. Recently, Mittal and Best (2008) examined the effect of different confinement geometries (e.g., planar, cylindrical, and spherical) on protein-folding thermodynamics and kinetics and found that the stabilization of the folded state relative to bulk conditions was independent of the geometry of confinement. Thus, here, we applied spherical cavity with different spherical radii *R*_S_ [e.g., 35 Å, 45 Å, and 55 Å designed based on Equation (2)] to mimic the crowding effects on the folding of MMTV pseudoknot over a wide range of Na^+^ concentrations [(Na^+^)]; see Figure 1A. Although the short-range repulsive interactions between atoms of macromolecules and the inner walls of sphere are often mimicked by the potential of ${\left(\frac{a}{r}\right)}^{12}$ (Klimov et al., 2002), since there is no significant difference between the potential and an ideal hard-wall potential, in this work, the interaction between the spherical cavity and the RNA pseudoknot was still approximate by a purely repulsive hard-wall potential for simplicity (Klimov et al., 2002; Tan and Chen, 2012):

(3) $$U_{RC}=\{\begin{array}{c} \;\infty \;if\;d>R_{s}-r\\ \;0\;if\;d\leq R_{s}-r\; \end{array},$$

where *d* is the distance between the CG beads and spherical cavity center, and *r* is the van der Waals radius of the CG beads.

### Simulation Algorithm

To improve the efficiency of conformational search, we used the Monte Carlo (MC)-simulated annealing algorithm with pivot move to predict the 3D structures for the MMTV pseudoknot at different conditions [e.g., temperature, (Na^+^) and radius of spherical confinement *R*_S_] from its sequence. The simulation is performed from high temperature (e.g., 400 K) to the target temperature (e.g., 298 K) with gradual cooling steps. At each temperature, RNA conformational changes are accomplished *via* the translations/pivot moves and the Metropolis criterion until the system reaches enough equilibrium (Shi et al., 2014a, 2018). The equilibrium conformations of the MMTV pseudoknot at each temperature under given conditions are saved to analyze the effect of spatial confinement on salt-dependent 3D structures, stability, and unfolding pathway of the RNA.

## Results and Discussion

To systematically evaluate how spatial confinement influence RNAs, we first employed the CG model to predict the 3D structures of the MMTV pseudoknot at different spatial confinements, ion conditions and temperatures. Afterward, we further examined the effect of spatial confinement on the stability and unfolding pathway of the pseudoknot over a wide range of ion concentrations.

### Effect of Spatial Confinement on Salt-Dependent Structures

First, based on the sequence, we predicted 3D structures of the MMTV pseudoknot at 25°C and 1 M [Na^+^]. As shown in Supplementary Figure 1, the system reaches enough equilibrium after a number of temperature-annealing MC steps (e.g., ~3 × 10^8^), and all the conformations in the following simulations are the structures predicted by the present model. The predicted mean radius of gyration *R*_g_ of the pseudoknot is ~15.7 Å, which is very close to that (15.1 Å) of the experimental structure (PDB code: 1rnk; https://www.rcsb.org), and the mean and minimum root-mean-square deviations (RMSDs) between predicted structure ensemble and experimental structure are ~4.6 and 3.2 Å, respectively, which suggest that the present model can give reliable prediction for the 3D structure of the RNA pseudoknot in the absence of spatial confinement; refer Shi et al. (2018).

**Figure 2:**
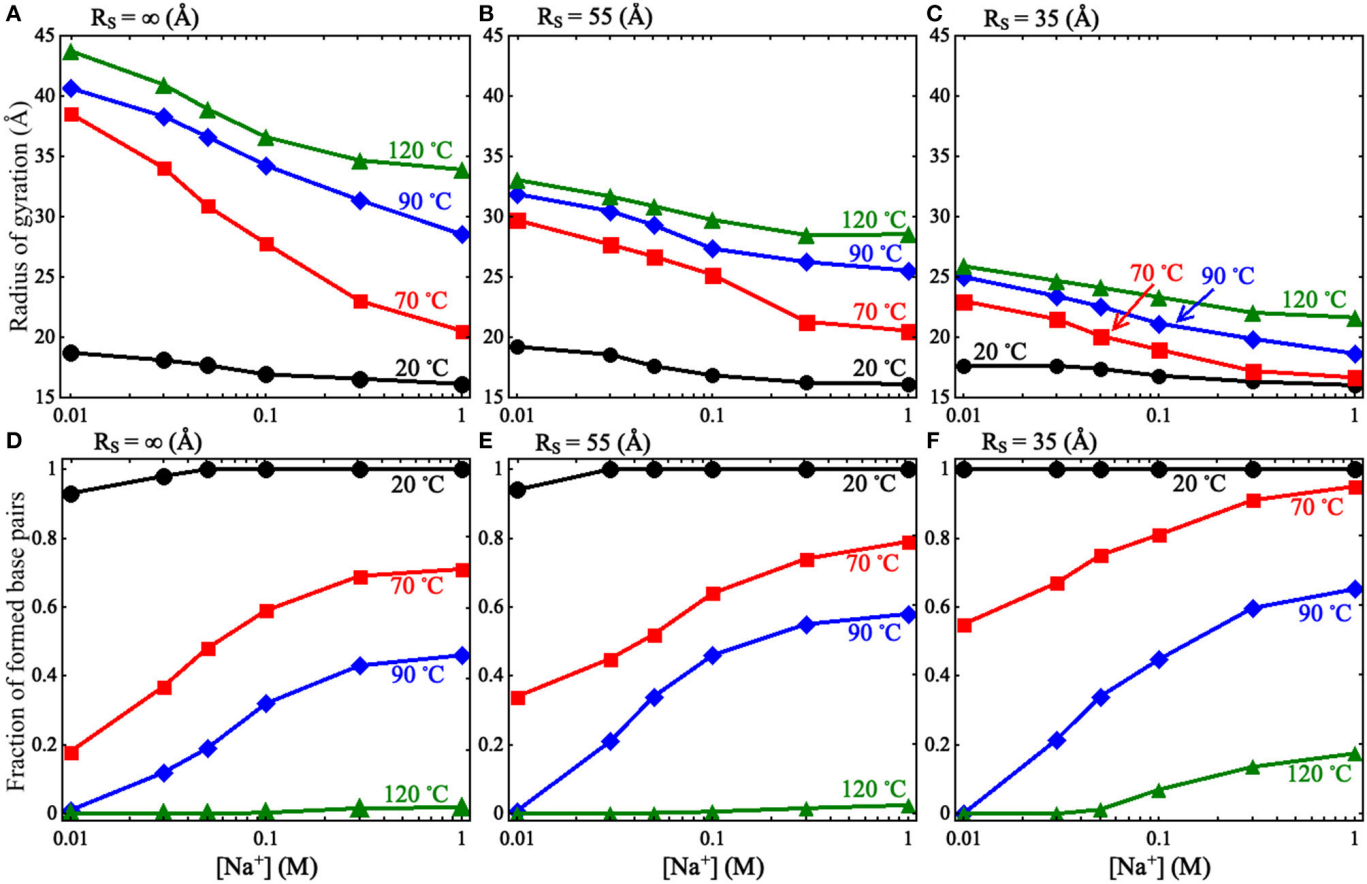
**(A–C)** The radii of gyration *R*_g_ as functions of [Na^+^] for the mouse mammary tumor virus (MMTV) pseudoknot at different temperatures and spatial confinements: **(A)** *R*_S_ = ∞ Å, **(B)** *R*_S_ = 55 Å, **(C)** *R*_S_ = 35 Å. **(D–F)** The fractions of formed base pairs as functions of [Na^+^] for the MMTV pseudoknot at different temperatures and spatial confinements: **(D)** *R*_S_ = ∞ Å, **(E)** *R*_S_ = 55 Å, and **(F)** *R*_S_ = 35 Å.

Afterward, we predicted 3D structures for the pseudoknot at different [Na^+^]s and temperatures under various spatial confinements (e.g., *R*_S_ from 35 to 55 Å). To obtain enough equilibrium conformations, we ran the simulations for a long time (e.g., ~7 × 10^8^ MC steps for *R*_S_ = ∞ Å); see Supplementary Figure 2. The calculated *R*_g_s and fractions *f* s of formed base pairs for the pseudoknot at different conditions are shown in Figure 2 and Supplementary Figure 2. Here, the fractions of formed base pairs *f* is calculated by:

(4) $$\begin{array}{c} f=\frac{\frac{1}{t}\sum_{t}N_{bp}(t)}{N_{bp}}, \end{array}$$

where *N*_*bp*_(*t*) is the total number of formed base pairs at MC step *t*, and *N*_*bp*_ is the number of predicted native base pairs at room temperature [e.g., ~10 bp at 1 M (Na^+^)].

**Figure 3:**
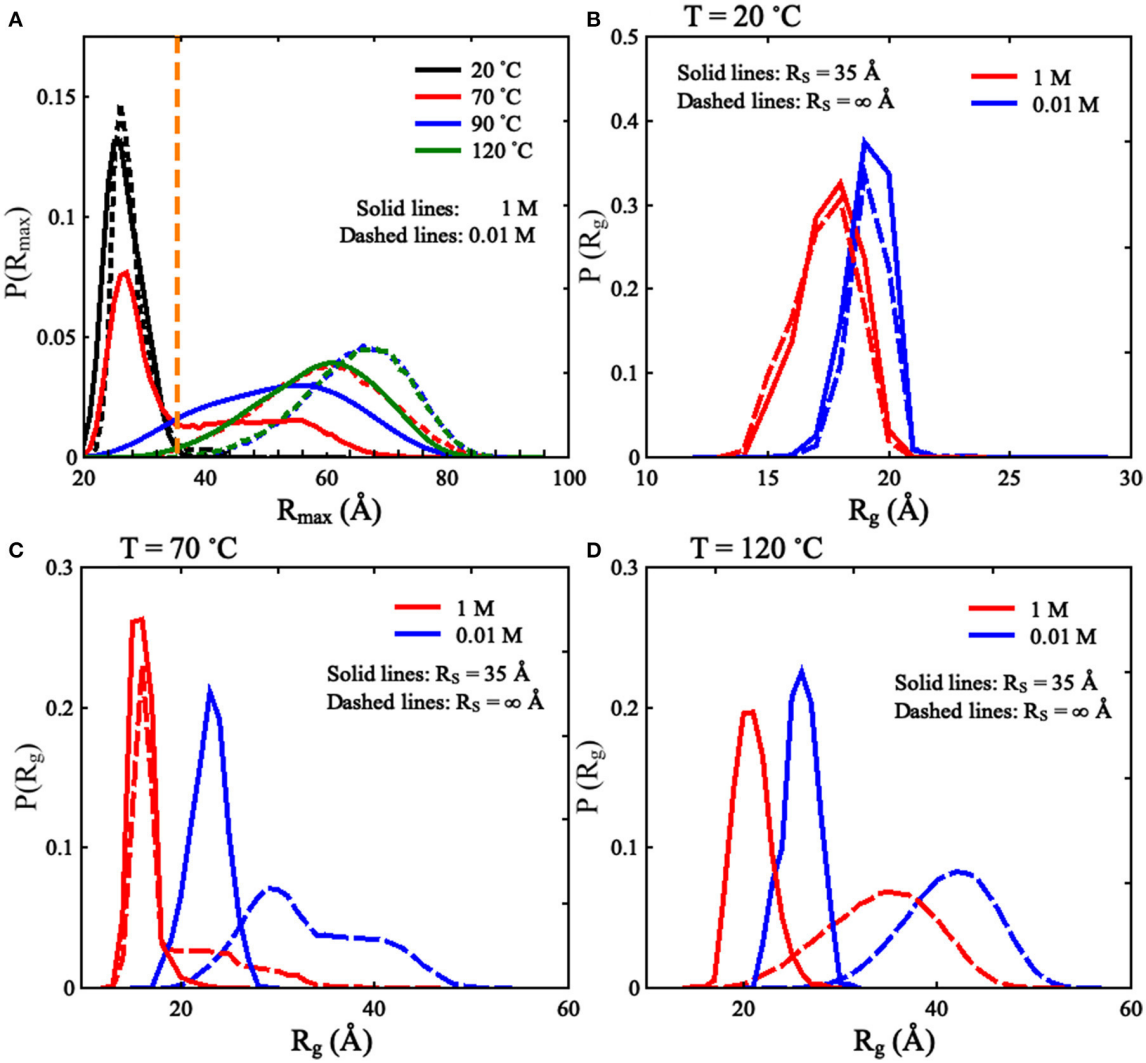
**(A)** The distributions of *R*_max_ [maximal distance between coarse-grained (CG) beads and their centroid] for the mouse mammary tumor virus (MMTV) pseudoknot at different temperatures and 1 M [Na^+^], and the vertical dashed line represents *R*_max_ of 35 Å. **(B–D)** The distributions of *R*_g_ for the conformations of the MMTV pseudoknot at different [Na^+^]s and temperatures: **(B)** 20°C, **(C)** 70°C, and **(D)** 120°C. In **(B–D)**, the solid lines and dashed lines represent *R*_S_ = 35 Å and *R*_S_ = ∞ Å, respectively.

As shown in Figure 2 and Supplementary Figure 2, we found that the spatial confinement has very slight effect on the folded structures at low temperature (e.g., 20°C), but has a considerable compacting effect on (partially) unfolded structures at high temperature (e.g., 120°C). For example, at 1 M [Na^+^] and 20°C, *R*_g_ of the RNA (~16.1 Å) within the spatial confinement of radius *R*_S_ = 35 Å is almost the same as that (~16.1 Å) without the spatial confinement, whereas at 120°C, the confinement of *R*_S_ = 35 Å can compact the structure of the RNA by ~12.3 Å in *R*_g_; see also in Supplementary Figure 4. This structure compacting effect is consistent with the recent works (Denesyuk and Thirumalai, 2011; Kilburn et al., 2013) and to directly understand this, we calculated the maximum distance *R*_max_ between CG beads and the centroid of the conformations at *R*_S_ = ∞ Å; see Figure 3. As shown in Figure 3A, the mean *R*_max_ for unfolded structures at 1 M [Na^+^] and 120°C is ~61 Å, which is much larger than confinement radii *R*_S_s used in this work (e.g., 35 Å), whereas *R*_max_ (~26 Å) for folded structures at 20°C is smaller than the used *R*_S_s. Thus, the spatial confinement can compact (partially) unfolded structures more significantly than folded ones, since (partially) unfolded structures are apparently more extended.

Furthermore, we examined the effect of spatial confinement on salt dependence of the pseudoknot structure compactness, since ions such as Na^+^ can promote RNA folding and favor folded compact structures (Draper et al., 2005; Tan and Chen, 2010, 2011; Lipfert et al., 2014). As shown in Figures 2A–C, the spatial confinement weakens the dependence of structure compactness on salt concentration over the wide range of temperatures, especially for (partially) unfolded structures. For example, in the absence of spatial confinement, the decrease of *R*_g_ due to the increase of [Na^+^] from 0.01 to 1 M are ~9.8 and 2.6 Å at 120 and 20°C, respectively, which are visibly larger than those (~4.2 and 1.6 Å at 120 and 20°C, respectively) in the presence of the confinement of *R*_S_ = 35 Å. This is because that the spatial confinement can spatially constraint the conformational space for an RNA inside, and such effect is more severe for more extended structures (e.g., at lower salt or at higher temperature); see also Figures 3B–D. Thus, the structure compactness of an RNA becomes more weakly dependent on salt concentration in a stronger spatial confinement.

Corresponding to the above-described effect on structure compactness, a spatial confinement would promote the formation of base pairs through its compacting effect as shown in Figures 2D–F. Such effect of promoting base pairing appears rather weak for the two limit cases of high salt/low temperature and low salt/high temperature, while it becomes apparent for other cases. At high salt/low temperature, the RNA is already folded and its structure is rather compact, and consequently a spatial confinement cannot cause the formation of more base pairs. For the other limit case of low salt/high temperature, the formation of base pairs would experience very strong electrostatic intra-chain repulsion and strong chain conformational entropy penalty, and the RNA chain would keep unfolded even in the presence of a spatial confinement; see Supplementary Figure 3. In the ranges of salt and temperature where the RNA is (partially) unfolded and the entropic penalty for base pairing/intra-chain electrostatic repulsion is not strong, a spatial confinement can have an apparent effect on promoting base pairing in the RNA.

### Effect of Spatial Confinement on Salt-Dependent Stability

We further examined the stability of the MMTV pseudoknot for different spatial confinements and ion conditions. During analyzing the unfolding of the pseudoknot, for each condition [i.e., (Na^+^) and *R*_S_], we performed one long-time simulation for the pseudoknot with enough conformations in equilibrium, and all the equilibrium conformations at different temperatures were used to analyze the RNA unfolding including melting temperatures and unfolding pathways; see Supplementary Figures 2–5. As shown in Supplementary Figures 5A,B, there are mainly three states that can be defined based on the number of base pairs at different temperatures: the fully folded pseudoknotted state (F), the intermediate hairpin states (I), and the unfolded coil state (U). And afterward, for each condition, the fractions of the F state and U state at different temperatures could be calculated, respectively, and fitted to a two-state model to obtain the two melting temperatures (*T*_m1_ and *T*_m2_) for the corresponding transitions (F→I and I→U); for more details, refer Supplementary Material and Shi et al. (2018).

**Figure 4:**
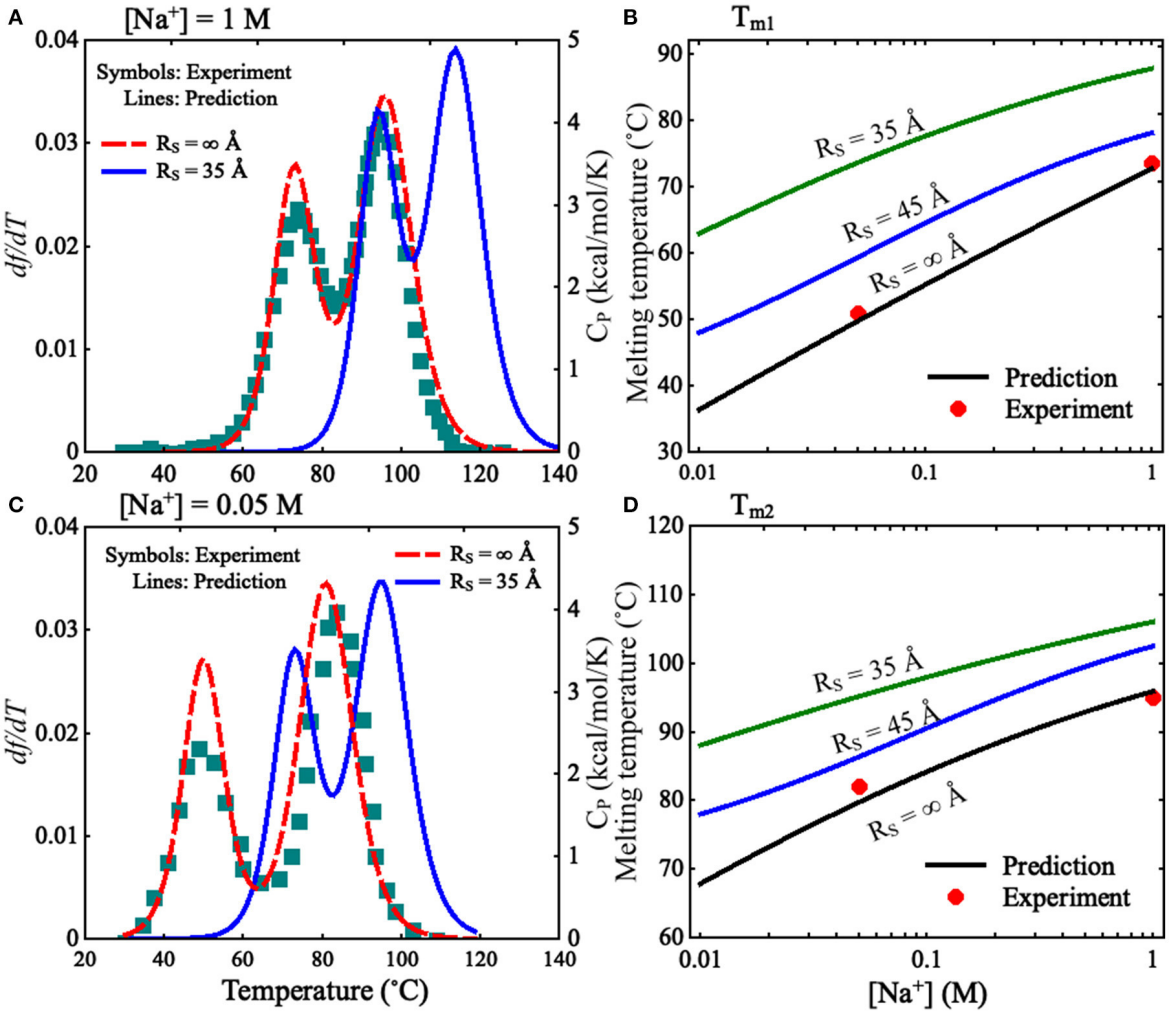
**(A,C)** The predicted and experimental melting profiles for the mouse mammary tumor virus (MMTV) pseudoknot at **(A)** 1 M [Na^+^] and **(C)** 0.05 M [Na^+^]. Lines: the predicted d*f* /d*T* (the first derivative of *f* with respect to temperatures) for *R*_S_ = ∞ Å (dashed line) and *R*_S_ = 35 Å (solid line), respectively. Symbols: the experimental heat capacity *C*_p_ for *R*_S_ = ∞ Å. **(B,D)** The melting temperatures *T*_m1_ **(B)** and *T*_m2_ **(D)** as functions of [Na^+^] for different *R*_S_. Symbols: the experimental data for *R*_S_ = ∞ Å; lines: the predicted *T*_m1_ and *T*_m2_ for *R*_S_ = ∞, 45 Å, and 35 Å.

**Figure 5:**
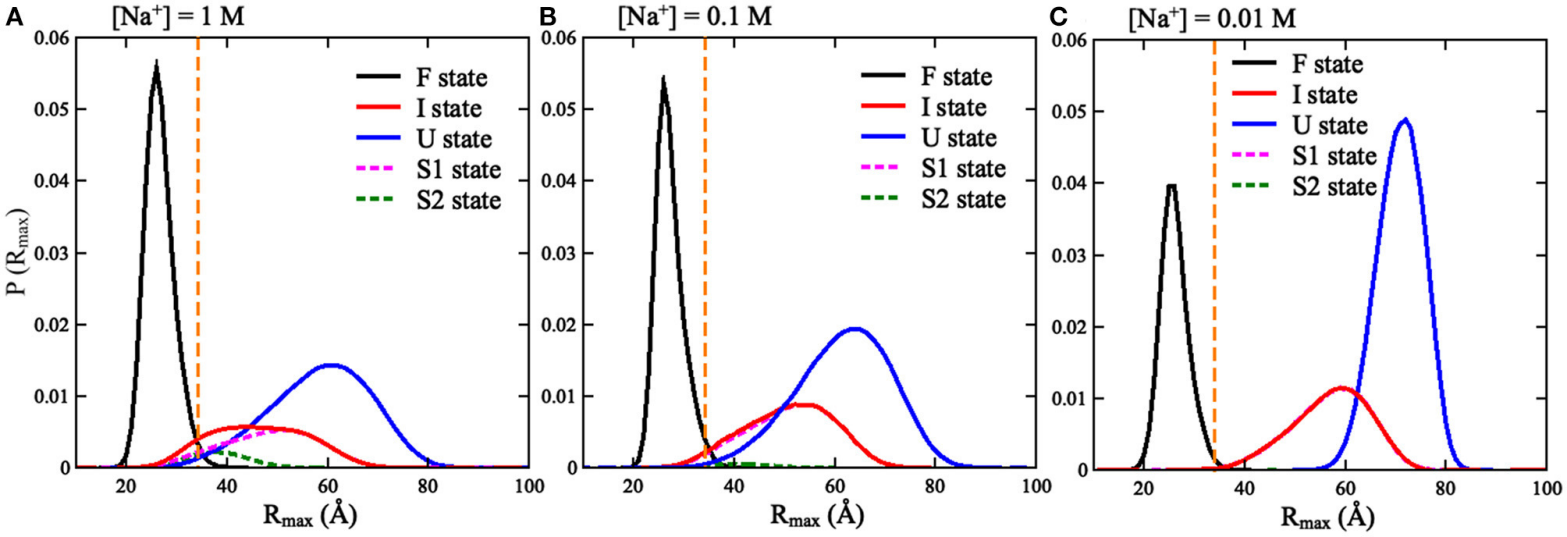
The distributions of *R*_max_ for the three states (F, I, and U) of the mouse mammary tumor virus (MMTV) pseudoknot over the conformations at all temperatures and different [Na^+^]'s: **(A)** 1 M [Na^+^], **(B)** 0.1 M [Na^+^], and **(C)** 0.01 M [Na^+^]. The vertical dashed lines in **(A–C)** represent *R*_max_ = 35 Å.

To validate the present model, we first predicted the melting temperatures for the pseudoknot at 1 M [Na^+^] and 0.05 M [Na^+^] in the absence of spatial confinement (i.e., *R*_S_ = ∞ Å) and then made the comparisons with the available experiment data (Shen and Tinoco, 1995). As shown in Figures 4B,D, the mean variation between the predicted and experimental *T*_m_s (*T*_m1_ and *T*_m2_) of the pseudoknot is <2°C, which indicates that the present model is reliable on predicting the stability of the pseudoknot at various salt concentrations. Figures 4B,D also shows that the stability of the pseudoknot can be significantly enhanced not only by the increase of [Na^+^] but also by the involvement of the spatial confinement. For example, the two melting temperatures (*T*_m1_ and *T*_m2_) of the pseudoknot increase from ~36 and 68°C to ~73 and 96°C, respectively, when [Na^+^] is increased from 0.01 to 1 M in the absence of spatial confinement, and they increase from ~73 and 96°C to ~88 and 106°C, respectively, when the spatial confinement with *R*_S_ = 35 Å is involved at 1 M [Na^+^]; see Figures 4B,D. Such salt-enhanced stability of the pseudoknot is attributed to the stronger ion neutralization effect at high salt (Tan and Chen, 2011; Shi et al., 2018). The enhancement effect of spatial confinement on the stability is because the spatial confinement can effectively limit the conformational space and such effect is more pronounced for the more extended unfolded/partially unfolded state (Denesyuk and Thirumalai, 2011), for example, *R*_max_ of the U state at 1 M [Na^+^] will shrink from ~61 to ≤35 Å as the spatial confinement (*R*_S_ = 35 Å) is involved; see Figure 5A.

We also calculated the increase of melting temperatures due to the involvement of the confinement of *R*_S_ = 35 Å for the two transitions of F→I and I→U, which are denoted by Δ*T*_m1,2_(*R*_S_ = 35 Å) = *T*_m1,2_(*R*_S_ = 35 Å)–*T*_m1,2_(*R*_S_ = ∞). Δ*T*_m1_(*R*_S_ = 35 Å) (~15.0°C) for the F→I transition is slightly larger than Δ*T*_m2_(*R*_S_ = 35 Å) (~10.0°C) for the I→U transition at 1 M [Na^+^]; see Figure 4 and Supplementary Figure 6. This indicates that the spatial confinement could enhance the stability of pseudoknotted state more strongly relative to corresponding intermediate (hairpin) state for the pseudoknot. This finding seems somewhat inconsistent with the recent study on the stability of the pseudoknot in human telomerase RNA under crowded conditions (Denesyuk and Thirumalai, 2011), and it is understandable. As shown in Figure 5A, for the pseudoknot at 1 M [Na^+^] without spatial confinement, we calculated the maximum distance *R*_max_ between CG beads and the centroid of each conformation. The distribution of *R*_max_ of the I state is only slightly more compact than that of the U state due to the small hairpin and very long tail in the I state, while it is significantly more extended than that of the F state. Thus, for the pseudoknot studied here, the spatial confinement can stabilize the folded state more strongly than the intermediate state through significantly reducing the volume accessible for the extended unfolded state and slightly less extended intermediate state.

Furthermore, we examined the effect of spatial confinement on the salt-dependent stability of the pseudoknot. As shown in Figures 4B,D, the salt dependence of the stability for the pseudoknot can be apparently weakened by the spatial confinement for both of the transitions. For example, the increase of *T*_m1_ (~25°C) due to the increase of [Na^+^] from 0.01 to 1 M for the confinement of *R*_S_ = 35 Å is apparently smaller than that of (~37°C) for *R*_S_ = ∞ Å, and similarly, the increase of *T*_m2_ (~18°C) with the increase of [Na^+^] from 0.01 to 1 M for the confinement of *R*_S_ = 35 Å is apparently smaller than that of (~28°C) for *R*_S_ = ∞ Å. Such weakened salt dependence of the stability by spatial confinement for the pseudoknot is understandable. Physically, the spatial confinement can restrict the conformations of RNAs, and such effect is more pronounced for more extended structures; see Figure 5. This causes that the unfolded U (or intermediate I) state becomes closer to the intermediate I (or folded F) state in structure compactness and consequently in charge density. Thus, the spatial confinement results in a weaker salt dependence of the stability of the pseudoknot for both of the F→I and I→U transitions due to the decreased differences in charge density between the F and I states and between the I and U states, respectively.

### Effect of Spatial Confinement on Salt-Dependent Unfolding Pathway

**Figure 6:**
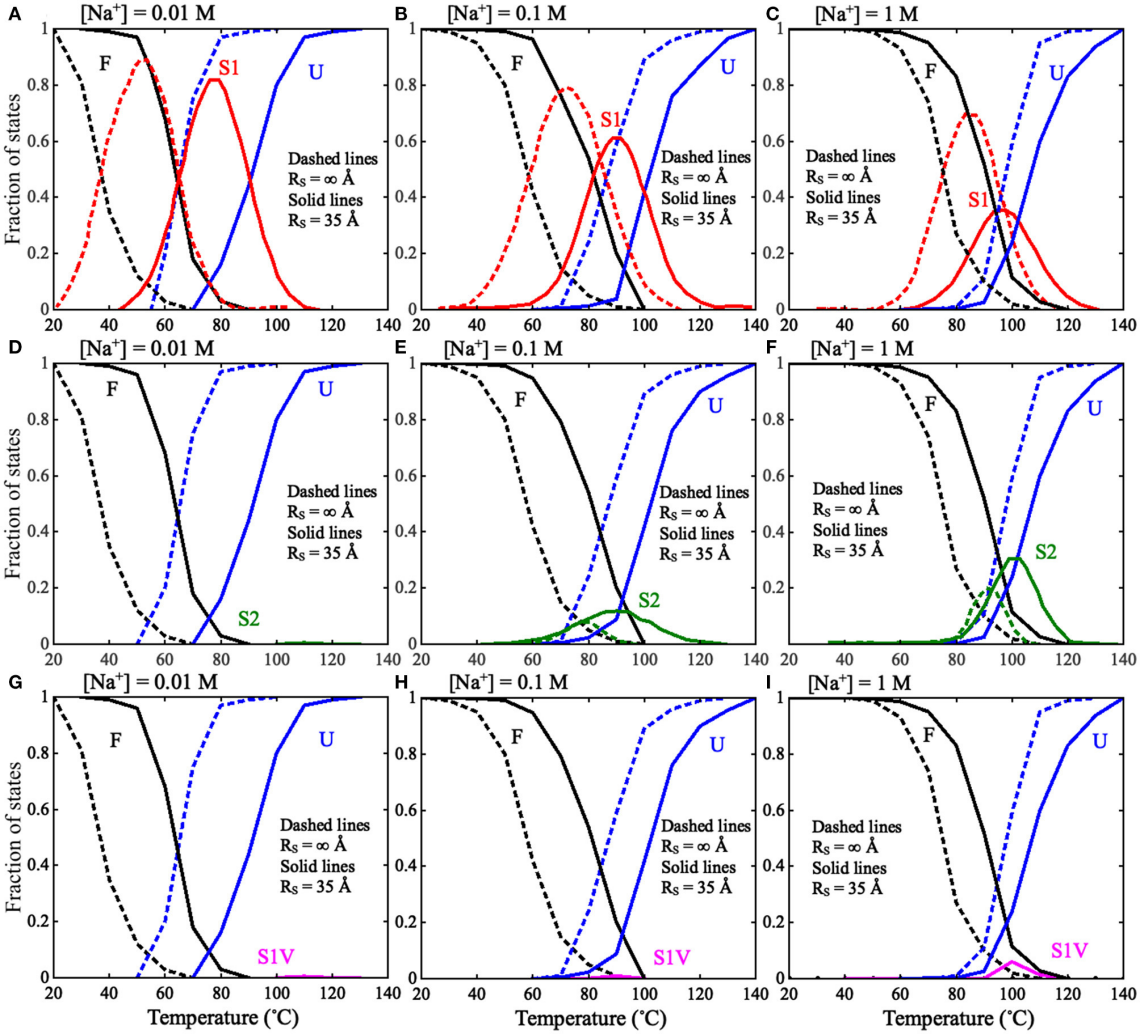
The fractions of F, S1, S2, S1V, and U states as functions of temperature in thermal unfolding of the mouse mammary tumor virus (MMTV) pseudoknot for *R*_S_ = ∞ Å (dashed lines) and *R*_S_ = 35 Å (solid lines): **(A–C)** The fractions of F, S1, and U states at **(A)** 0.01 M [Na^+^], **(B)** 0.1 M [Na^+^], and **(C)** 1 M [Na^+^]. **(D–F)** The fractions of F, S2, and U states at **(D)** 0.01 M [Na^+^], **(E)** 0.1 M [Na^+^], and **(F)** 1 M [Na^+^]. **(G–I)** The fractions of F, S1V, and U states at **(A)** 0.01 M [Na^+^], **(B)** 0.1 M [Na^+^], and **(C)** 1 M [Na^+^]. F stands for fully folded RNA, S1 stands for hairpin intermediate with Stem 1, S2 stands for hairpin intermediate with Stem 2, S1V stands for Stem1 variation, and U stands for fully unfolded RNA.

**Figure 7:**
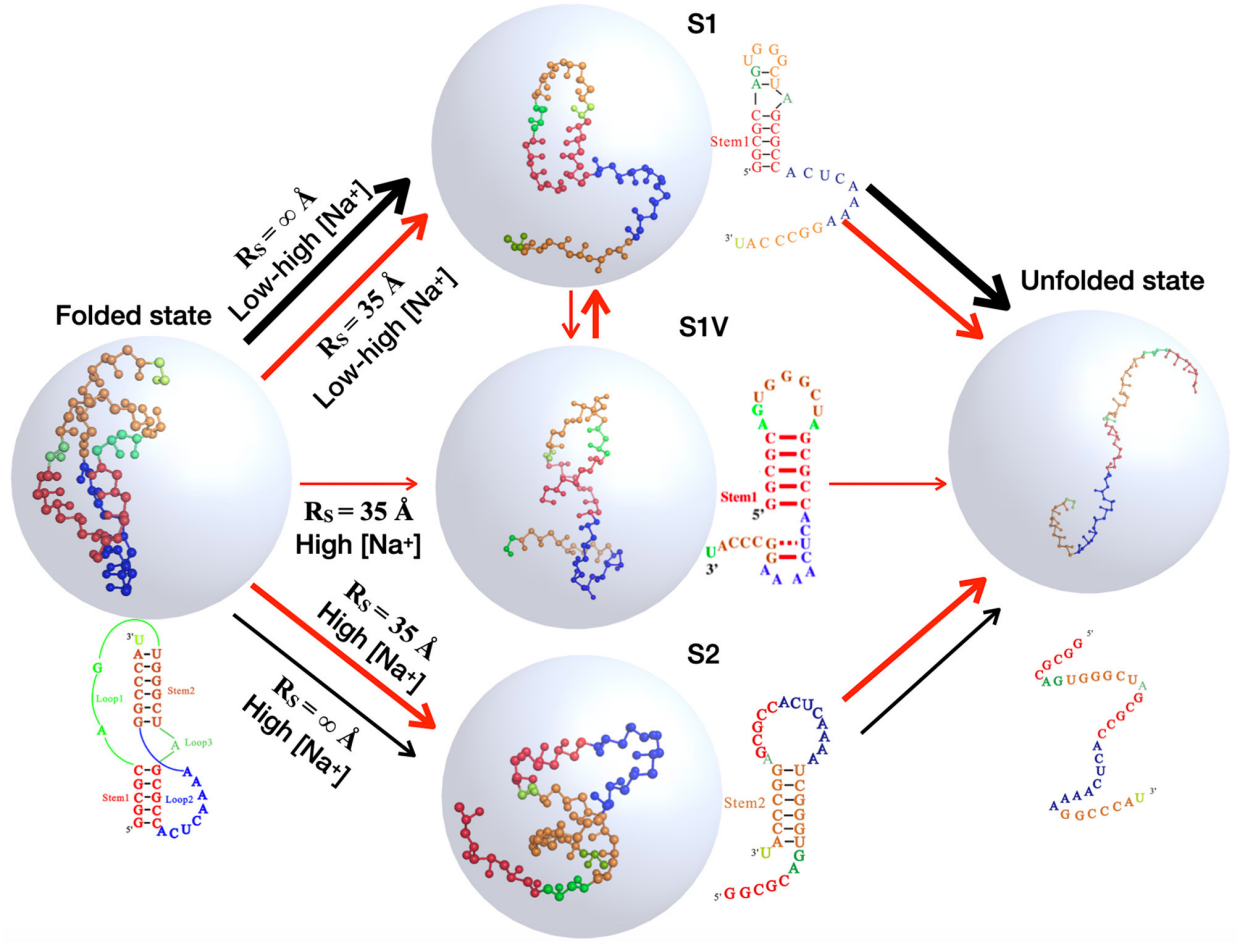
The schematic diagrams show the structural transitions of the mouse mammary tumor virus (MMTV) pseudoknot in ion solutions along the unfolding pathway inferred from the respective fractions of states shown in Figure 6, for the two typical cases: in the absence of spatial confinement (*R*_S_ = ∞ Å) and in the presence of strong spatial confinement (*R*_S_ = 35 Å). Ball-stick: the predicted 3D structures for different states shown with the PyMol (http://www.pymol.org).

Because of the importance of the unfolding pathway of RNA pseudoknots (Roca et al., 2018; Shi et al., 2018), we made further comprehensive analyses for the pseudoknot at different conditions to examine the effect of spatial confinement on the unfolding pathway. Based on the simulations for the pseudoknot at a given (temperature and salt) condition, the I state can also be divided into two independent hairpin states: S1 and S2, with only Stem 1 or Stem 2, respectively; see Figure 1, and the fractions of S1 and S2 states can also be calculated at any condition including salt condition and spatial confinement. As shown in Figure 6 for the factions of different states and Figure 7 for the illustration of unfolding pathway, in the absence of spatial confinement (*R*_S_ = ∞ Å), the maximum fractions of the S1 and S2 states are ~70 and 20% at 1 M [Na^+^], respectively, and changes into ~89 and 0% when [Na^+^] is decreased from 1 M to 0.01 M. This indicates that the pseudoknot has two unfolding pathways at high salt: the major one of F→S1→U and the minor one of F→S2→U, which can be dramatically modulated into the only one pathway of F→S1→U by low salt. Such prediction agrees well with the recent experiments (Shen and Tinoco, 1995; Roca et al., 2018), and the unfolding pathway is determined by the relative stability between the S1 and S2 states, which can be changed by ions due to the different sizes of hairpins in S1 and S2 states (Shi et al., 2018). As shown in Figure 7, compared with the S1 state, the S2 state has a larger hairpin and consequently becomes less stable with the decrease of [Na^+^] due to the stronger intra-chain electrostatic repulsion at lower [Na^+^] (Tan and Chen, 2007, 2011).

Furthermore, as shown in Figures 6, 7, a spatial confinement can also greatly influence the unfolding pathway of the pseudoknot at high salt concentrations, for example, at 1 M [Na^+^], the maximal fractions of S1 and S2 states change from ~70 and 20% to ~35 and 30% when the spatial confinement with *R*_S_ of 35 Å is introduced; see Figures 6C,F. Such effect of spatial confinement is reasonable. As shown in Figure 5A, there is a significant difference in *R*_max_ distribution between two intermediate (S1 and S2) hairpin states at 1 M [Na^+^]. The mean of *R*_max_ of S1 state (~52 Å) is apparently larger than that of S2 state (~37 Å) due to the longer tail in the S1 state. The result indicates that the S1 state with large size could be more strongly suppressed by the spatial confinement than the S2 state, and consequently, the fraction of S1 state would be reduced by the spatial confinement. In contrast, the S2 state, with the majority of *R*_max_ close to the radius of spherical cavity (i.e., *R*_S_ = 35 Å), could be weakly suppressed by the spatial confinement and accordingly, the fraction of the S2 state can increase due to the apparent decreased fraction of the other intermediate (S1) state. Very importantly, the spatial confinement brings an apparent appearance of intermediate state of S1 state with additional base pairs in tail (e.g., U_22_-G_29_ and C_23_-G_28_). Here, we defined this new state including Stem 1 and additional base pairs in 3′-end as the S1V state, which can be considered a variant of S1 state. As shown in Figure 6, the spatial confinement of *R*_S_ = 35 Å causes the increase of the fraction of S1V state from ~0.9 to 6% at 1 M [Na^+^]. The apparent appearance of S1V state is attributed to the compacting effect on the long tail of S1 state by the spatial confinement. In contrast to high salt, the spatial confinement has little effect on the unfolding pathway at low salt, and there is still only unfolding pathway of F→S1→U at low salt even in the presence of the spatial confinement of *R*_S_ = 35 Å. This is because the strong intra-chain electrostatic repulsion counteracts the effect of spatial confinement at low salt.

The above analyses for the pseudoknot indicate that the unfolding pathway of RNAs generally could be modulated by both ion conditions and spatial confinement. In Figure 7, we summarized the unfolding pathway of the pseudoknot in the presence of spatial confinement. At low salt, the pseudoknot has only one unfolding pathway in the presence/absence of spatial confinement. However, at high salt, the spatial confinements cause two major unfolding pathways with similar fractions and a new minor pathway through the S1 variant, in contrast to one major and one minor pathways in the absence of spatial confinement.

## Conclusions

Motivated by the correlations between crowded cell environments and functions of RNA molecules, we examined the effect of spatial confinement on the salt dependence of 3D structure, thermal stability, and unfolding pathway for the MMTV pseudoknot using our previously developed CG model for RNAs. First, based on the predicted 3D structures of the pseudoknot under different spatial confinements from its sequence, we found that the spatial confinement can weaken the dependence of 3D structure compactness on salt through severely restricting the extended RNA conformations at low ion concentrations or at high temperatures. Second, the spatial confinement can enhance the thermal stability of the RNA structures, and such enhancement is more pronounced for low salt, causing that the spatial confinement can apparently weaken the dependence of stability on salt for both the pseudoknotted and intermediate hairpin states. This is attributed to the stabilizing effect of the spatial confinement is more pronounced for more extended (partially) denatured states at low salt. Finally, the unfolding pathway of the pseudoknot can be modulated by both ion conditions and spatial confinements. At low salt, the pseudoknot has only one unfolding pathway in the presence/absence of spatial confinement. However, at high salt, the spatial confinements cause two major unfolding pathways with similar fractions and a new minor pathway, in contrast to one major and one minor pathways in the absence of spatial confinement.

Although we made reliable predictions and comprehensive analyses on salt-dependent structure stability for the MMTV pseudoknot under different spatial confinements, the effect of spatial confinement on structure stability for extensive RNA pseudoknots with various sequences/lengths and functional RNAs with complex structures (e.g., tRNA and riboswitches) in ion solutions should be studied by further developing the present CG model (Zhang et al., 2011; Kilburn et al., 2016; Yamagami et al., 2018), and further development on the present model may need to take into account the tertiary contacts beyond the canonical and wobble base pairs already involved in the model (Weinreb et al., 2016; Jian et al., 2019; Wang and Zhao, 2020). Furthermore, there are some other physical and chemical factors that could affect how crowding modulates RNA stability, which is beyond the approximation of the spatial confinement considered here (Zhou et al., 2008; Nakano et al., 2009; Paudel and Rueda, 2014; Strulson et al., 2014; Leamy et al., 2016, 2017; Feig et al., 2017). For example, recent studies showed that molecular crowders could not only stabilize the tertiary structures of macromolecules by excluded volume but also destabilize RNA secondary structures through the surface interactions and reduction in water activity (Zhou et al., 2008; Gao et al., 2016; Leamy et al., 2017). Moreover, the related works from Thirumalai et al. also indicated that small crowders can increase the stability of compact structures of human telomerase RNA to a greater extent than larger ones (Denesyuk and Thirumalai, 2011), and the crowders with attractive interactions with RNA bases can stabilize an RNA hairpin more than inert crowding agents of the same size (Pincus et al., 2008). In addition, the interaction between ions (especially multivalent ion) and RNAs can be influenced by crowders (Yu et al., 2016), which is beyond the implicit ion treatment presented here and is required to be addressed in the future work (Tan and Chen, 2007, 2012; Xu and Chen, 2020). Thus, to effectively simulate the RNA folding in the cellular environment with ions and different crowding macromolecules is still an important issue required to be solved. Nevertheless, this work could be very helpful for understanding the salt-dependent structure and stability for RNAs in cellular-like confined environment.

## Data Availability Statement

The original contributions presented in the study are included in the article/Supplementary Material, further inquiries can be directed to the corresponding author/s.

## Author Contributions

Z-JT, Y-ZS, and CF designed the research. CF and Y-ZS performed the simulations. Z-JT, CF, Y-LT, and Y-XC analyzed the data. CF, Y-ZS, and Z-JT wrote the manuscript. All authors discussed the results and reviewed the manuscript.

## Conflict of Interest

The authors declare that the research was conducted in the absence of any commercial or financial relationships that could be construed as a potential conflict of interest.
