# Supplementary material for: Salt-Dependent RNA Pseudoknot Stability: Effect of Spatial Confinement
Source: Front Mol Biosci. 2021 Apr 13;8:666369. doi: 10.3389/fmolb.2021.666369 (PMC8078894; doi:10.3389/fmolb.2021.666369)
Supplement: Supplementary file 1 [file Data_Sheet_1.pdf]

Supplementary Material

1 The energy terms in the coarse-grained force field and parameters

The coarse-grained (CG) force field consists of a variety of terms based on physical interactions or derived from the regularities seen in experimental structures. These energy terms can be classified into two categories: bonded potentials and nonbonded potentials.

1.1 Bonded potentials

The bonded potential U_{bonded} contains the bond length energy U_b, bond angle energy U_a and dihedral angle energy U_d, which are used to describe the local connectivity between CG beads:

$$U_{bonded} = U_b + U_a + U_d; \tag{S1}$$

where

$$U_b = \sum_{bonds} K_b\,(r - r_0)^2; \tag{S2}$$

$$U_a = \sum_{angles} K_\theta\,(\theta - \theta_0)^2; \tag{S3}$$

$$U_b = \sum_{dihedrals} \left\{K_\varphi[1 - \cos(\varphi - \varphi_0)] + \frac{1}{2}K_\varphi[1 - cos3(\varphi - \varphi_0)]\right\}. \tag{S4}$$

Here, K_b, K_θ, and K_φ denote the energy strength, and r_0, θ_0, and φ_0 represent the corresponding values at energy minimum. These parameters were extracted from statistical analyses of the experimental structures from the Protein Data Bank (PDB) (http://www.rcsb.org/pdb/hom/home.do), following by a fine adjustment based on comparisons between predicted results and corresponding experimental data (Shi et al., 2014). It should be pointed out that two sets of parameters $Para_{\text{helical}}$ and $Para_{\text{nonhelical}}$ were obtained from the single-strands/loops and the stems in experimental structures, respectively (Shi et al., 2014). The $Para_{\text{nonhelical}}$ is used in folding process to well simulate RNA folding approximated as a free chain and the $Para_{\text{helical}}$ is used only for CG beads of base-paired nucleotides in structure refinement process to depict the more standard geometry of helical parts; see more details in Refs. (Shi et al., 2014, 2015, 2018). And, the final parameters of bonded potentials (Eqs. S2-S4) are listed in Table S1.

Table S1. The parameters of bonded potentials in Eqs. S2-S4.

	Bond U_b			
	K_b (kcal/mol/Å^2)		r_0 (Å)	
	$Para_{\text{helical}}$ [a]	$Para_{\text{nonhelical}}$ [b]	$Para_{\text{helical}}$ [a]	$Para_{\text{nonhelical}}$ [b]
P_iC_i	133.4	98.2	3.95	3.95
C_iP_{i+1}	75.0	42.5	3.93	3.93
C_iN_i	85.6	24.8	3.35	3.45
	Angle U_a			
	K_θ (kcal/mol/rad^2)		θ_0 (rad)	
	$Para_{\text{helical}}$ [a]	$Para_{\text{nonhelical}}$ [b]	$Para_{\text{helical}}$ [a]	$Para_{\text{nonhelical}}$ [b]
$P_iC_iP_{i+1}$	18.3	9.3	1.74	1.75
$C_{i-1}P_iC_i$	43.9	21.3	1.76	1.78
$P_iC_iN_i$	35.5	9.7	1.63	1.64
$N_iC_iP_{i+1}$	99.8	15.2	1.66	1.66
	Dihedral U_d			
	K_φ (kcal/mol/rad^2)		φ_0 (rad)	
	$Para_{\text{helical}}$ [a]	$Para_{\text{nonhelical}}$ [b]	$Para_{\text{helical}}$ [a]	$Para_{\text{nonhelical}}$ [b]
$P_iC_iP_{i+1}C_{i+1}$	2.8	1.1	2.56	2.51
$C_{i-1}P_iC_iP_{i+1}$	10.5	4.3	-2.94	-2.92
$C_{i-1}P_iC_iN_i$	3.8	0.8	-1.16	-1.18
$N_{i-1}C_{i-1}P_iC_i$	4.2	0.7	0.88	0.78

[a] The $Para_{\text{helical}}$ are only used in the processes of folded structure refinement for the base-pairing regions (stems) in the initially folded structure. [b] The $Para_{\text{nonhelical}}$ are used in RNA folding processes to possibly describe RNAs as free chains.

1.2 Nononded potentials

The nonbonded potential $U_{nonbonded}$ contains the base-pairing energy U_{bp}, base-stacking energy U_{bs}, coaxial-stacking energy U_{cs}, electrostatic energy U_{el} and excluded volume U_{exc}, which were used to accurately describe the 3D structure predictions for RNAs:

$$U_{nonbonded} = U_{bp} + U_{bs} + U_{cs} + U_{el} + U_{exc}, \tag{S5}$$

where

$$U_{bp} = \sum_{i<j-3}^{N_{bp}} \frac{\varepsilon_{bp}}{1 + k_{NN}(r_{N_iN_j} - r_{NN})^2 + k_{CN}\sum_{i(j)}(r_{C_iN_j} - r_{CN})^2 + k_{PN}\sum_{i(j)}(r_{P_iN_j} - r_{PN})^2}; \tag{S6}$$

$$U_{bs} = \sum_{i,j}^{N_{st}} |G_{i,i+1,j-1,j}| \left\{ \left[5\left(\frac{\sigma_{st}}{r_{i,i+1}}\right)^{12} - 6\left(\frac{\sigma_{st}}{r_{i,i+1}}\right)^{10} \right] + \left[5\left(\frac{\sigma_{st}}{r_{j,j-1}}\right)^{12} - 6\left(\frac{\sigma_{st}}{r_{j,j-1}}\right)^{10} \right] \right\}; \tag{S7}$$

$$U_{cs} = \sum_{i,k,l,j}^{N_{cst}} |G_{i,k,l,j}| \left\{ \left[1 - e^{-a(r_{ik} - r_{cs})}\right]^2 + \left[1 - e^{-a(r_{lj} - r_{cs})}\right]^2 - 2 \right\}; \tag{S8}$$

$$U_{el} = \sum_{i<j}^{N_P} \frac{(Qe)^2}{4\pi\varepsilon_0\varepsilon(T)r_{ij}} e^{-r_{ij}/l_D}; \tag{S9}$$

$$U_{exc} = \sum_{i<j}^{N} \begin{cases} 4\varepsilon\left[\left(\frac{\sigma_0}{r_{ij}}\right)^{12} - \left(\frac{\sigma_0}{r_{ij}}\right)^{6}\right] & if\ r_{ij} \le \sigma_0 \\ 0 & if\ r_{ij} \ge \sigma_0 \end{cases}. \tag{S10}$$

In Eq. S5, U_{bp} is the orientation-dependent base-pairing interaction between bases in the canonical Watson-Crick base pairs (G-C and A-U) and the wobble base pairs (G-U), and the interaction strength $\varepsilon_{AU} = \varepsilon_{GU} = 0.5\varepsilon_{GC}$. r_{NN}, r_{CN}, and r_{PN} are three distances (between CG beads in paired nucleotides) obtained from the known structures to determine whether the base-pair is well formed, and k_{NN}, k_{CN} and k_{PN} are the corresponding energy strength. It should be noted that the U_{bp} can only be calculated when the distance $r_{N_iN_j}$ between two base beads N_i and N_j satisfies the base-paired criteria: 8.6Å $\le r_{N_iN_j} \le$ 9.3Å, which is defined based on the statistics of known structures. U_{bs} is the base-stacking interaction between two nearest neighbour base pairs, and the sequence-dependent strength of base-stacking energy $G_{i,i+1,j-1,j}$ can be estimated from the combination of the experimental thermodynamics parameters (Xia et al., 1998) and Monte Carlo algorithm; more details can be found in Refs. (Shi et al., 2014, 2015, 2018). σ_{st} in Eq. S7 is the optimum distance of two neighbour bases in the known helix structures. The coaxial-stacking energy U_{cs} in Eq. S5 describes

the energy between the base-pairs at the interface of two discontinuous neighbor helices, and $G_{i-j,k-l}$ is the sequence-dependent base-stacking strength, which is approximated as the stacking strength between the corresponding nearest-neighbor base-pairs in an uninterrupted helix (Xia et al., 1998). In Eq. S8, r_{ik} (or r_{jl}) is the distance between two interfaced bases i(j) and k(l) of two stems, a represents the extent of distance constraint, and r_{cs} is the optimum distance between two coaxially stacked stems in known structures in PDB (Shi et al., 2014).

U_{el} in Eq. S5 represents electrostatic interaction between phosphate groups (P) with reduced charges Qe, which is accounted for by the Debye-Hückel approximation combined with the concept of counterion condensation; see Eq. S9 (Manning, 1978). In Eq. S9, r_{ij} is the distance between two charged beads i and j, and ε_0 and $\varepsilon(\mathrm{T})$ are the permittivity of vacuum and the effective dielectric constant, respectively. l_D in Eq. S9 is Debye length

$$l_D = \left(\frac{\varepsilon_0 \varepsilon(T) k_B T}{2 N_A e^2 I}\right)^{1/2}, \tag{S11}$$

which describes the ionic screening. Here, k_B is the Boltzmann constant, T is the absolute temperature in Kelvin, and $I = \frac{1}{2}\sum_i c_i z_i^2$ is the ionic strength which is dependent on the ion concentration and ion valences of salt solution. Based on the Manning's counterion condensation theory (Manning, 1978), the reduced charge fraction Q could be written as $Q = \frac{b}{l_B}$ for pure monovalent ion solutions, where b (was taken as 5.5 Å) is the charge spacing on RNA backbone and l_B is the Bjerrum length. For a mixed Na^+/Mg^{2+} ion solution, we assume $Q = f_{\mathrm{Na}^+} Q_{\mathrm{Na}^+} + (1 - f_{\mathrm{Na}^+}) Q_{\mathrm{Mg}^{2+}}$, where f_{Na^+} and $(1 - f_{\mathrm{Na}^+})$ represent the contribution fractions from Na^+ and Mg^{2+}, respectively. f_{Na^+} can be approximately calculated by the empirical formula previously derived by the tightly bound ion model which could account for the Na^+-Mg^{2+} competition in stabilizing RNAs (Tan and Chen, 2007, 2011),

$$f_{\mathrm{Na}^+} = \frac{[\mathrm{Na}^+]}{[\mathrm{Na}^+] + x[\mathrm{Mg}^{2+}]}, \tag{S12}$$

where $x = (8.1 - 64.8/N)(5.2 - \ln[\mathrm{Na}^+])$. $[Na^+]$ and $[Mg^{2+}]$ are the corresponding bulk concentrations in molar (M), and N is the chain length; see more details in Refs. (Shi et al. 2015; Tan and Chen, 2007). The last term in Eq. S5 is the non-bonded excluded volume interaction between the CG beads. In Eq. S10, ε = 0.26 kcal/mol is the interaction strength, σ_0 is the sum of the radii of bead i and j, and r_{ij} is the distance between bead i and j.

The parameters for the nonbonded potentials are listed in Table S2, which can also be found in our related works (Shi et al., 2014, 2015, 2018; Jin et al., 2018, 2019).

Table S2. The parameters for the nonbonded potentials in Eqs. S6-S10.

r_{NN}(Å)	8.9	k_{NN}(Å^{-2})	3.6
r_{CN}(Å)	12.2	k_{CN}(Å^{-2})	1.9
r_{PN}(Å)	13.9	k_{PN}(Å^{-2})	0.7
σ_{st}(Å)	4.8	$\varepsilon_{bp}(kcal/mol)$	-3.5
r_{cs}(Å)	5.0	$a(kcal/mol/$Å$)$	0.4

2 The calculation of the melting temperatures

To investigate the stability of the MMTV pseudoknot, we predicted the 3D structures for the pseudoknot at different temperatures using the present model, and then, calculated the melting temperatures based on the fractions of different states. For example, Fig. S5A shows that the number of formed base pairs changes at different temperatures for the pseudoknot at 1M [Na^+], and there are mainly three states across the full range of temperature: the folded native state (F) at low temperature, the unfolded random state (U) at high temperature and the partially unfolded intermediate hairpin states (I) at medium temperature. Here, the F state is defined as the formation of all the two stems (Stem 1 and Stem 2), and the U state is referred to the coil state with no stable base pairs, while the other conformations besides the F and U ones are classified as the I state. Since the base pairs in the ends of stems are generally unstable, each stem is defined by the formation of three or more non-terminal base pairs. For example, the Stem1 is determined by the formation of the middle three base pairs (G_2-C_{18}, C_3-G_{17}, and G_4-C_{16}). Then, the fractions of the three states at each temperature can be calculated and the fractions of folded and unfolded states ($f_F(T)$ and $f_U(T)$) could be fitted to a two-state model through the following equations (Shi et al., 2018):

$$f_F(\mathrm{T}) = \frac{1}{1 + e^{(\mathrm{T}-\mathrm{T_{m1}})/\mathrm{dT_1}}}; \qquad (S11)$$

$$f_U(\mathrm{T}) = 1 - \frac{1}{1 + e^{(\mathrm{T}-\mathrm{T_{m2}})/\mathrm{dT_2}}}; \qquad (S12)$$

where T_{m1} and T_{m2} are the two melting temperatures of the corresponding transitions (F → I and I → U), respectively. dT_1 and dT_2 are the corresponding adjustable parameters (Shi et al., 2018). Furthermore, based on the $f_{\mathrm{F}}(\mathrm{T})$ and $f_{\mathrm{U}}(\mathrm{T})$ the fraction of the number of denatured base pairs f can be calculated through the following equation (Shi et al., 2018):

$$f = 1 - \left[(1 - f_I) \cdot f_F(\mathrm{T}) + f_I \cdot \left(1 - f_U(\mathrm{T})\right)\right] \qquad (S13)$$

Here, f_I is the fraction of the number of denatured base pairs when the fraction for I state is maximum. Based on the Eq. S13, the df/dT (the first derivative of f with respect to temperature) profile can be calculated to compare with the corresponding experimental data.

3 Supplementary Figures

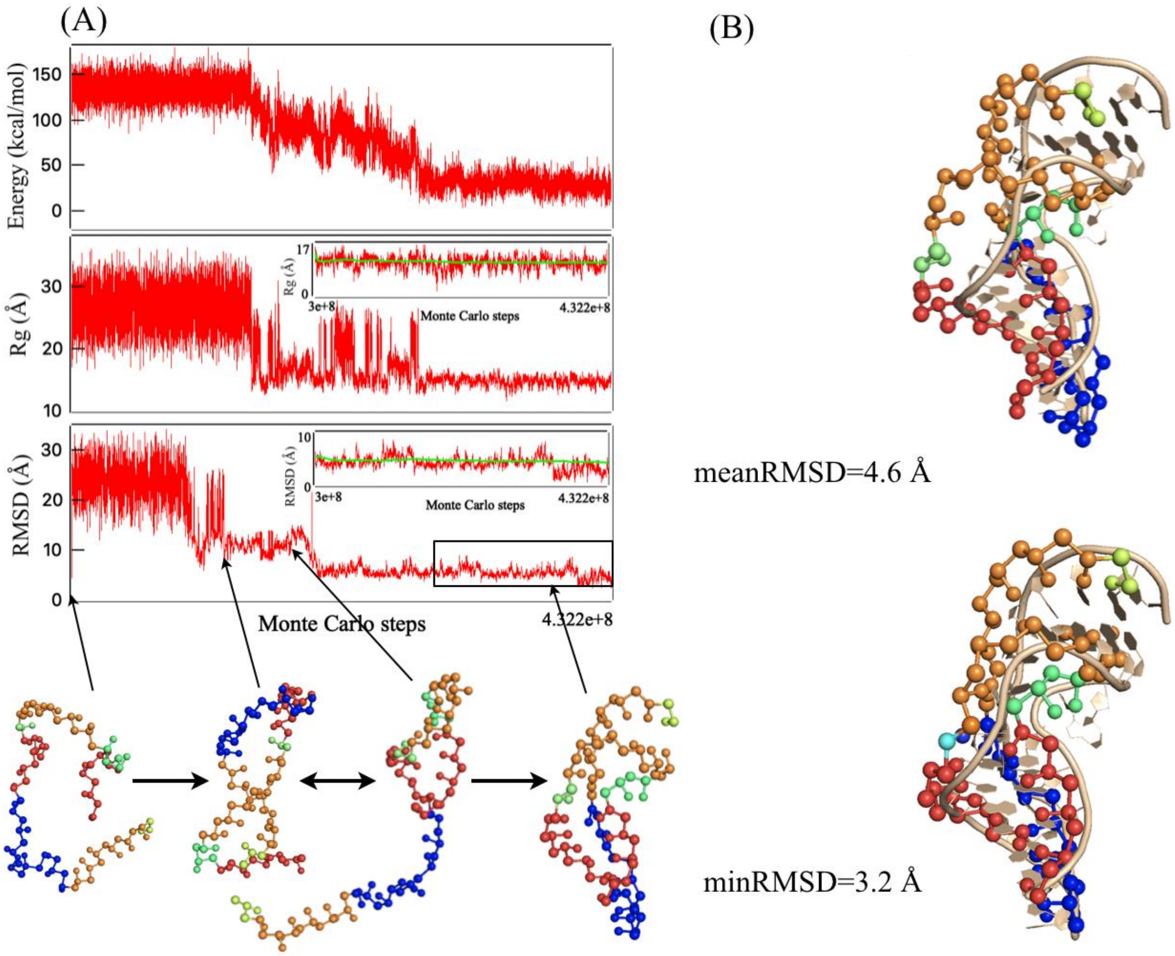

Figure S1. (A) The time-evolutions of the energy (top panel), the radius of gyration R_g (middle panel), the RMSDs between predicted structures and the native structure in PDB (bottom panel), and the typical 3D structures during the Monte Carlo simulated annealing simulation of the MMTV pseudoknot (PDB code: 1rnk). (B) The predicted 3D structures (ball-stick) with the mean RMSD (top) and the minimum RMSD (bottom) for the MMTV pseudoknot from its native structure (cartoon). The mean and minimum RMSDs are 4.6 Å and 3.2 Å, respectively. The RMSDs are calculated between the P, C, N beads in the predicted structures and the corresponding P, C4’, N1/N9 atoms in the native structure. The 3D structures are shown with the PyMol (http://www.pymol.org).

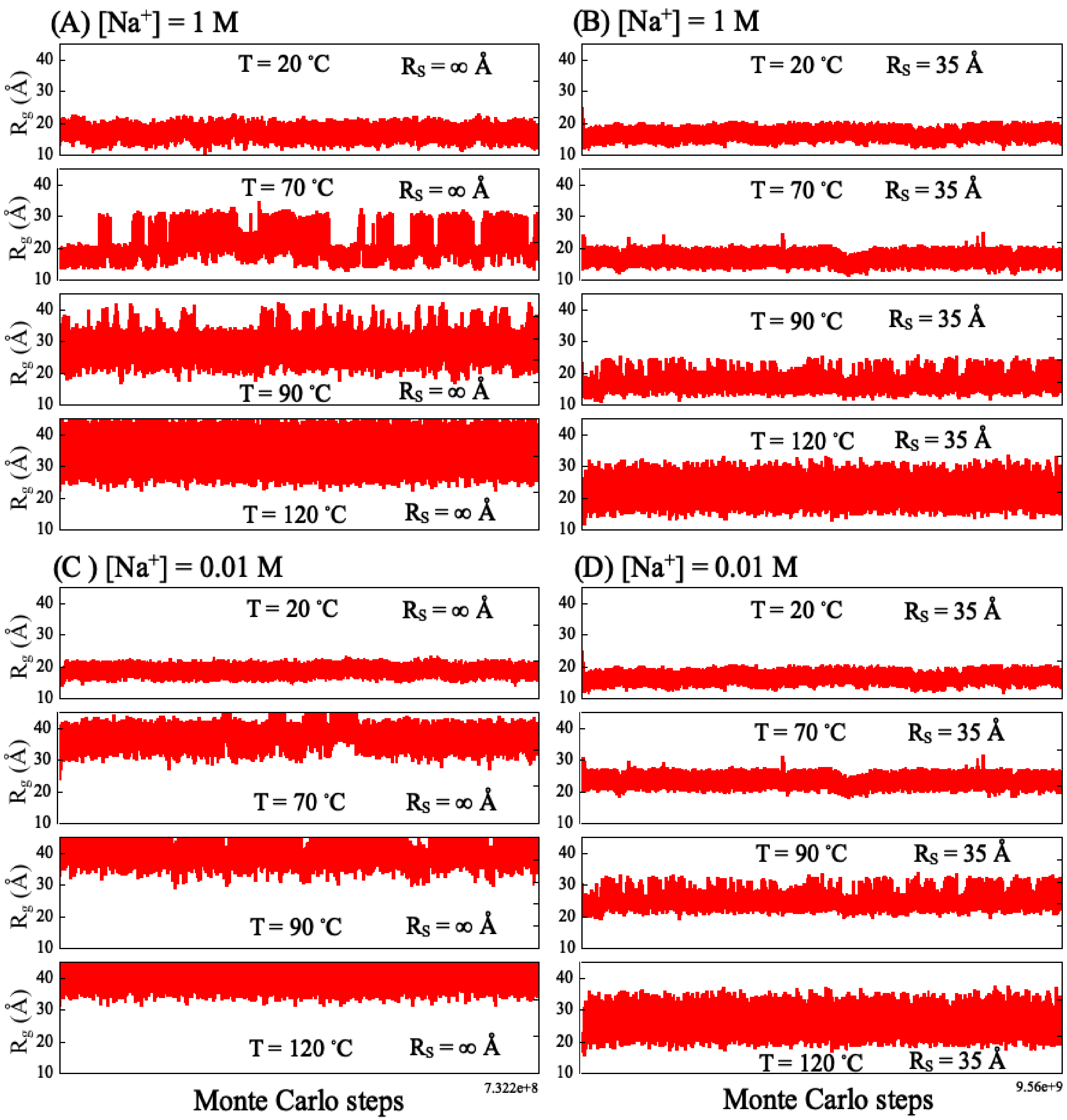

Figure S2. The time-evolutions of the radius of gyration R_g for the MMTV pseudoknot at different conditions: (A) $[Na^+]$ = 1M and $R_S = \infty$ Å; (B) $[Na^+]$ = 1M and R_S = 35 Å; (C) $[Na^+]$ = 0.01M and $R_S = \infty$ Å; (D) $[Na^+]$ = 0.01M and R_S = 35 Å. The temperatures in (A-D) are 20 °C, 70 °C, 90 °C, and 120 °C from top to bottom, respectively.

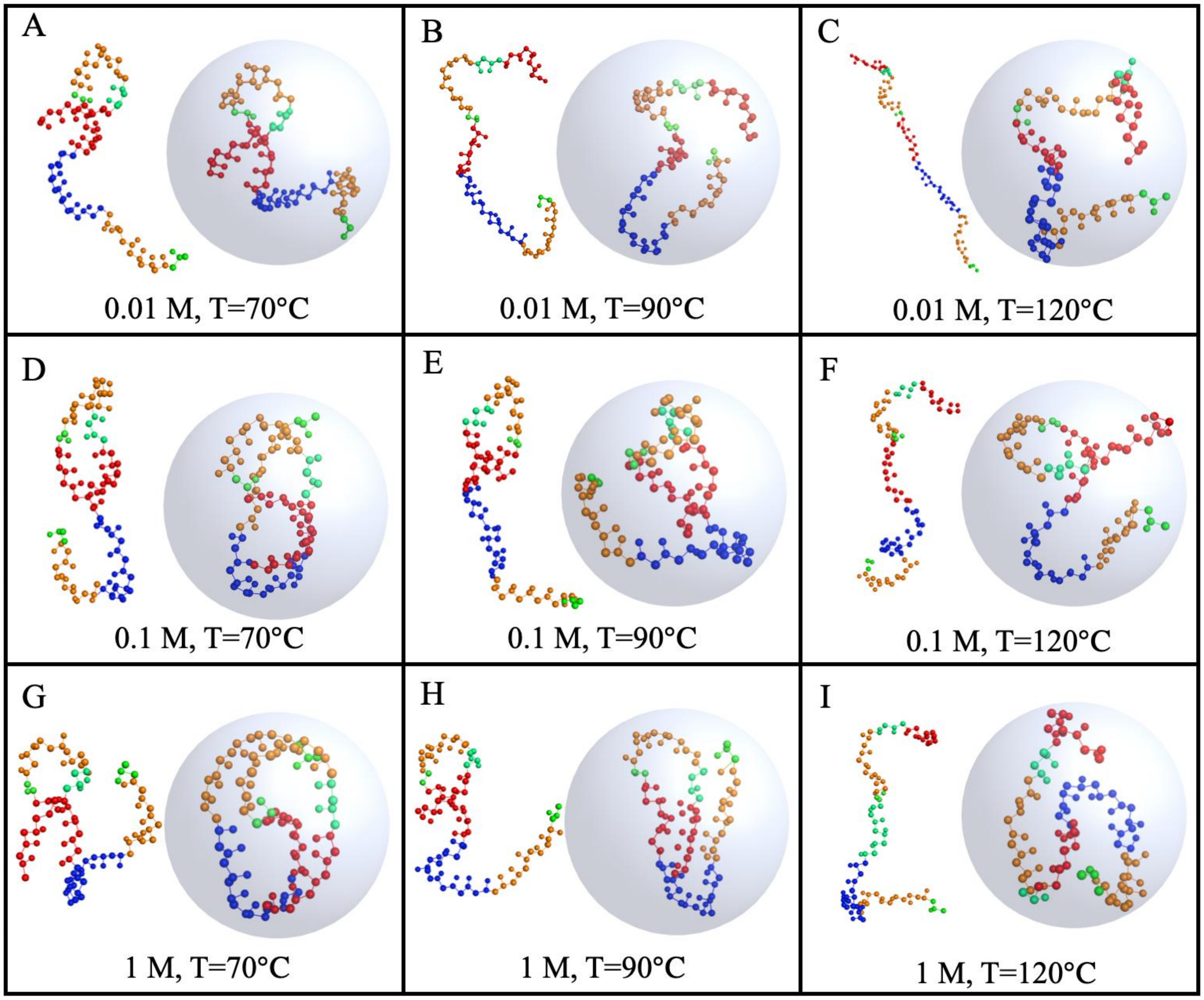

Figure S3. The predicted 3D structures of the MMTV pseudoknot at different conditions. The salt concentrations and temperatures are shown in respective panels. The structures in the shaded spherical cavity and in free space denote those in the spatial confinement of R_S=35 Å and those in the absence of spatial confinement ($R_S = \infty$ Å)

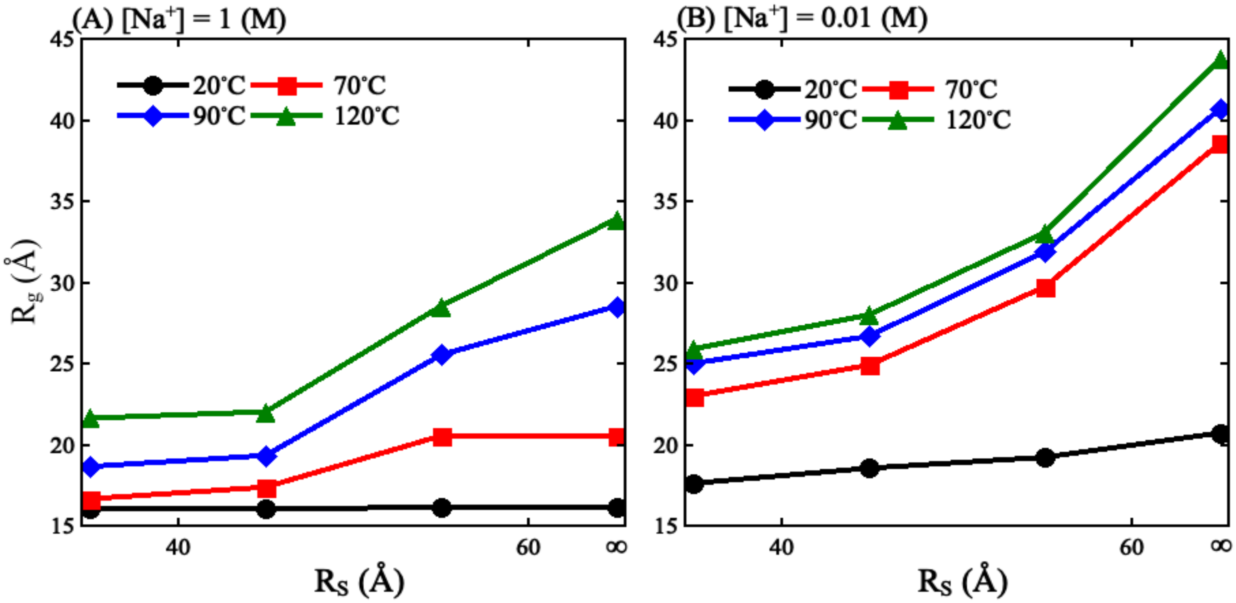

Figure S4. (A, B) The averaged radius of gyration (R_g) of the predicted 3D structures for the MMTV pseudoknot as a function of R_S at different temperatures. (A) $[Na^+]$=1M; (B) $[Na^+]$=0.01M.

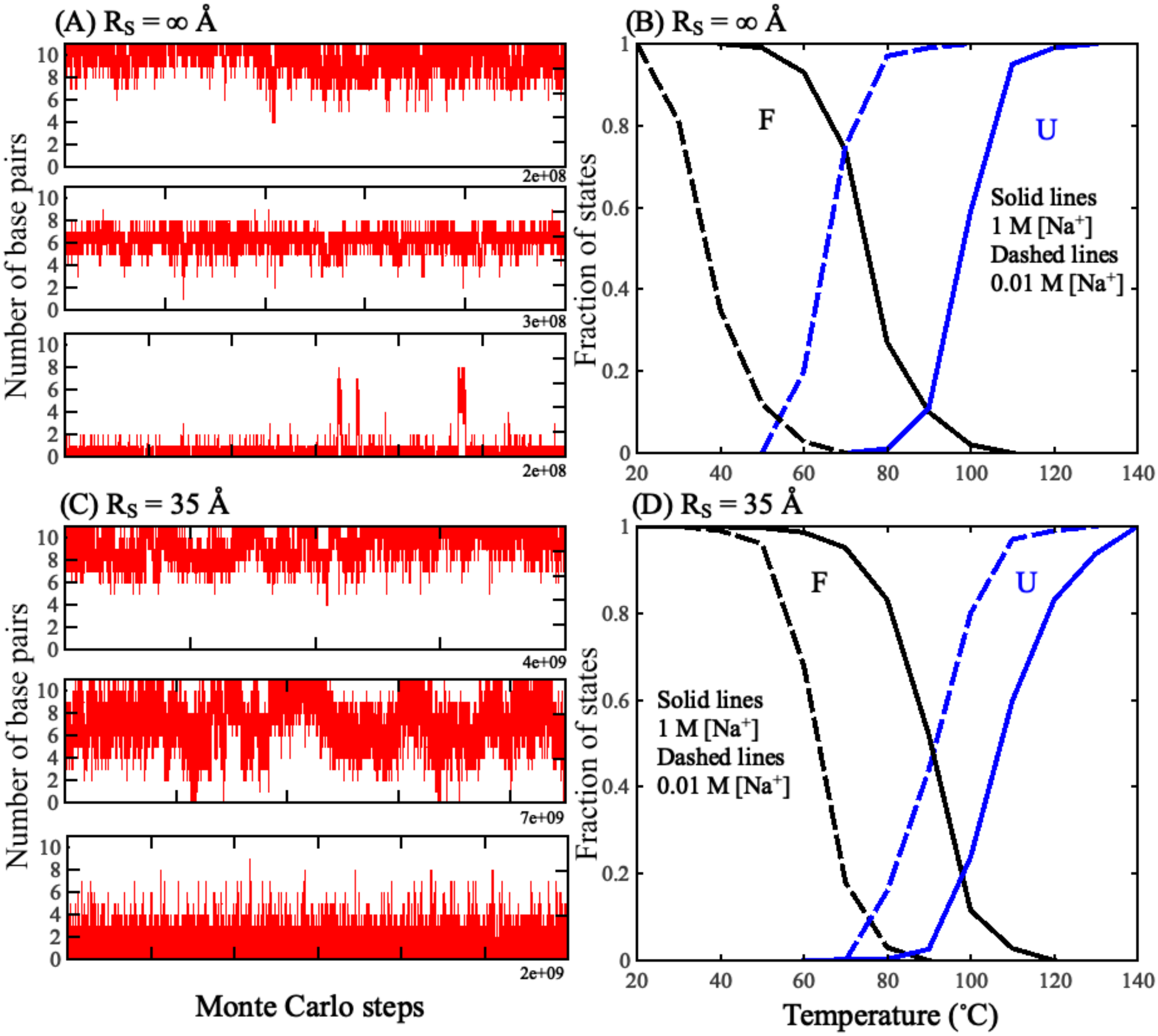

Figure S5. The stability prediction for the MMTV pseudoknot in the present model. (A,C) The time-evolutions of the number of base pairs at different temperatures (20 °C, 80 °C, and 120°C; from top to bottom) and different spatial confinements: (A) $R_S = \infty$ Å and (C) R_S = 35 Å. (B,D) The fractions of the folded state (F, black), and the unfolded state (U, blue) as functions of temperature for the MMTV pseudoknot at (C) $R_S = \infty$ Å and (D) R_S = 35 Å. Solid lines: 1M [Na^+]. Dashed lines: 0.01M [Na^+].

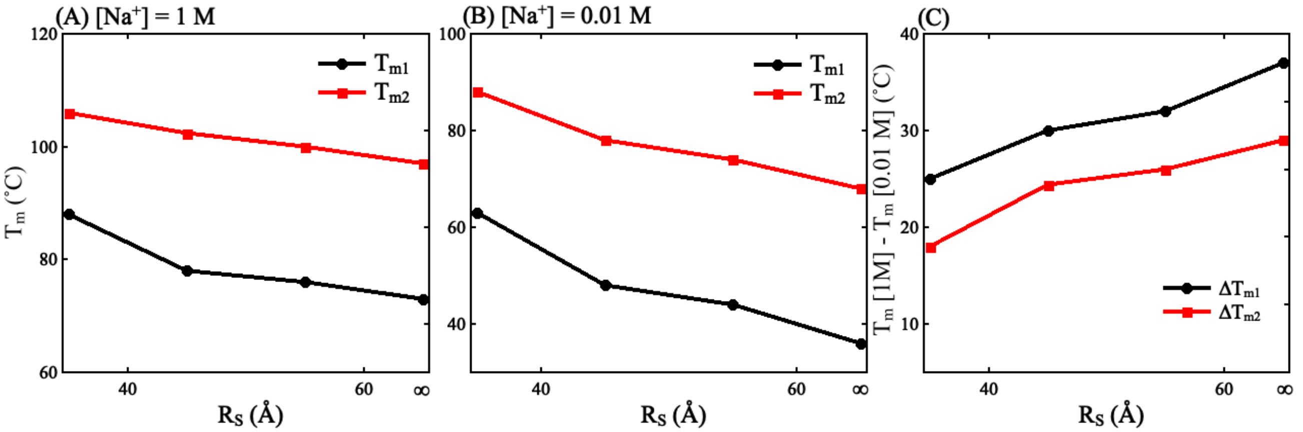

Figure S6. (A, B) The melting temperatures T_{m1} and T_{m2} as functions of R_{S} at (A) $[\mathrm{Na}^+]$ =1M and (B) $[\mathrm{Na}^+]$=0.01M, respectively. (C) The increase of two melting temperatures (T_{m1} and T_{m2}) from 0.01 M $[\mathrm{Na}^+]$ to 1 M $[\mathrm{Na}^+]$ ($\Delta T_m = T_m([\mathrm{Na}^+ = 1\mathrm{M}]) - T_m([\mathrm{Na}^+ = 0.01\mathrm{M}])$) as a function of R_{S}.

Supporting References

Jin, L., Shi, Y. Z., Feng, C. J., and Tan, Z. J. (2018). Modeling structure, stability, and flexibility of double-stranded RNAs in salt solutions. Biophys. J. 115,1403-1416.

Jin, L., Tan, Y. L., Wu, Y., Wang, X., Shi, Y. Z., and Tan, Z. J. (2019). Structure folding of RNA kissing complexes in salt solutions: predicting 3D structure, stability, and folding pathway. RNA, 25, 1532-1548.Manning, G. S. (1978). The molecular theory of polyelectrolyte solutions with applications to the electrostatic properties of polynucleotides. Q. Rev. Biophys.11, 179–246.

Manning, G. S. (1978). The molecular theory of polyelectrolyte solutions with applications to the electrostatic properties of polynucleotides. Q. Rev. Biophys.11, 179–246.

Shi, Y. Z., Jin, L., Feng, C. J., Tan, Y. L., and Tan, Z. J. (2018). Predicting 3D structure and stability of RNA pseudoknots in monovalent and divalent ion solutions. PLoS. Comput. Biol. 14, e1006222.

Shi, Y. Z., Jin, L., Wang, F. H., Zhu, X. L., and Tan, Z. J. (2015). Predicting 3D structure, flexibility, and stability of RNA hairpins in monovalent and divalent ion solutions. Biophys. J. 109, 2654-2665.

Shi, Y. Z., Wang, F. H., Wu, Y. Y., and Tan, Z. J. (2014). A coarse-grained model with implicit salt for RNAs: predicting 3D structure, stability and salt effect. J. Chem. Phys. 141,105102.

Tan, Z. J., and Chen, S. J. (2007). RNA helix stability in mixed Na^{+}/Mg^{2+} solution. Biophys. J. 92, 3615-3632.

Tan, Z. J., and Chen, S. J. (2011). Salt contribution to RNA tertiary structure folding stability. Biophys. J. 101, 176.

Xia, T., SantaLucia, J., Burkand, M. E., Kierzek, R., Schroeder, S. J., Jiao, X., Cox, C., and Turner, D. H. (1998). Thermodynamic parameters for an expanded nearest-neighbor model for formation of RNA duplexes with Watson-Crick base pairs. Biochemistry 37, 14719–14735.
